# A 3D Scanning System for Inverse Analysis of Moist Biological Samples: Design and Validation Using Tendon Fascicle Bundles

**DOI:** 10.3390/s20143847

**Published:** 2020-07-10

**Authors:** Sylwia Dabrowska, Martyna Ekiert, Kaja Wojcik, Marek Kalemba, Andrzej Mlyniec

**Affiliations:** Faculty of Mechanical Engineering and Robotics, AGH University of Science and Technology, Al. Mickiewicza 30, 30-059 Cracow, Poland; dabrows@agh.edu.pl (S.D.); mekiert@agh.edu.pl (M.E.); kwojcik@agh.edu.pl (K.W.); mkalemba@agh.edu.pl (M.K.)

**Keywords:** non-contact measurement, three-dimensional imaging, tendon, tendon fascicle bundles, 3D model, soft tissues

## Abstract

In this article, we present the design and validation of a non-contact scanning system for the development of a three-dimensional (3D) model of moist biological samples. Due to the irregular shapes and low stiffness of soft tissue samples, the use of a non-contact, reliable geometry scanning system with good accuracy and repeatability is required. We propose a reliable 3D scanning system consisting of a blue light profile sensor, stationary and rotating frames with stepper motors, gears and a five-phase stepping motor unit, single-axis robot, control system, and replaceable sample grips, which once mounted onto the sample, are used for both scanning and mechanical tests. The proposed system was validated by comparison of the cross-sectional areas calculated based on 3D models, digital caliper, and vision-based methods. Validation was done on regularly-shaped samples, a wooden twig, as well as tendon fascicle bundles. The 3D profiles were used for the development of the 3D computational model of the sample, including surface concavities. Our system allowed for 3D model development of samples with a relative error of less than 1.2% and high repeatability in approximately three minutes. This was crucial for the extraction of the mechanical properties and subsequent inverse analysis, enabling the calibration of complex material models.

## 1. Introduction

Biomedical imaging is an integral component in the study of soft tissue biomechanics. Advancement in three-dimensional (3D) imaging techniques of various biological tissues (e.g., tendons, muscle, skin) allows enhanced understanding of their physiology, structure, and biomechanical properties. Morphological changes in the complex hierarchical structure of tissue [1] caused by disease, aging, biological calcification, or injury [2,3,4,5] contribute to various alterations in the mechanical and material properties of these tissues [6,7,8]. The mechanical properties of the tissue, such as viscoelasticity [9] and the resulting stress distribution, are related to its dimensions and geometric structure. Therefore, it is necessary to analyze the shape and structure of biological samples from these tissues to determine their mechanical behavior and fully understand the relationship between structure and function in these tissues. Accurate measurement of specimen shape is crucial in the determination of its mechanical properties, since an incorrectly assumed geometry generates significant discrepancies in the material parameters extracted from experiments. Determination of material model parameters such as elastic modulus, ultimate strength, yield stress, stress relaxation, and other viscoelastic properties based on tensile or fatigue tests requires knowledge of the cross-sectional area (CSA) or shape of the whole sample in the case of inhomogeneous deformation of the specimen. A variety of techniques for the measurement of CSA and specimen geometry have been developed thus far. Such techniques include destructive, non-destructive contact, and non-contact methods. Destructive methods have successfully been implemented to determine CSA [10,11] and geometry, but the need to destroy the sample disqualifies this approach in the estimation of material model parameters based on the inverse approach. Non-destructive contact techniques are prone to errors due to the required contact with the sample [12], which can deform the surface of soft tissue. Non-contact techniques offer meaningful advantages with respect to the repeatability and reliability of soft tissue measurements. The lack of physical contact with the sample ensures the accuracy and repeatability of the measurements. One of the most precise non-contact techniques is laser micrometry developed by Lee and Woo [13,14]. However, this technique has one major disadvantage: the geometry affects the imaging results; thus, laser micrometers are unable to detect concavities in the sample. The concavities are flat during image reconstruction, which produce a less accurate CSA measurement in complex shapes. Moreover, laser micrometers cannot be used for 3D geometry reconstruction of the sample for subsequent inverse analysis. Other techniques that can be used for characterizing the 3D structure of soft tissues include laser and photographic scanners. The laser scanning device, described by Heuer et al. [15], can be used for surface digitalization of soft tissue or whole musculoskeletal structures and subsequent estimation of the strain field. The photographic scanner, proposed by Hashemi et al. [16], allows CSA calculation at any point along the length of the specimen, as well as the creation of a 3D model from the collection of 2D images. However, this device cannot detect surface concavities as well as a laser scanning device. Other measurement techniques use the structured white light [17,18] or charge-coupled device (CCD) laser displacement sensors [19,20,21,22] in the determination of tissue morphological properties. These methods are characterized by good repeatability and can capture the entire geometry of the sample. However, they are limited to samples with a CSA greater than 10 mm^2^. Geometric changes and deformation of the sample during loading can be assessed using vision-based 3D digital image correlation (3D DIC) techniques [23,24,25,26,27,28]. Three-dimensional DIC can be useful in the measurement of both external surface deformation of samples and deformation of samples in the bulk. Obuchowicz et al. [29] used the DIC method to analyze a series of photographs of the specimen surface, as well as a series of ultrasound images acquired in a specified time interval during tissue relaxation. It is not necessary to apply speckle patterns when analyzing ultrasound images with DIC, which is an advantage of this technique. However, the analysis of the specimen surface requires the application of the special contrasting speckle pattern [25]. Most existing solutions used for the imaging of tendon or tendon subcomponent samples do not detect surface concavities and/or do not have the required accuracy; thus, they cannot be used for the reconstruction of the Sample 3D geometry. Methods that do provide the required repeatability and accuracy of imaging do not allow for rapid measurement (within a matter of minutes) of samples already mounted in the grips, which would prevent sample dehydration. Maintaining unchanged boundary conditions during tissue scanning and testing, as well as adequate tissue hydration is crucial in the process of parameter estimation using inverse analyses as described below. Three-dimensional imaging is an important tool for analyzing the shapes and movements of soft tissues. In clinical practice, tendon dimensions are often used in the identification of injury or in the planning of tendon surgery. A variety of techniques for the measurement of CSA and specimen geometry have been developed thus far. Clinically available measurement techniques are very important for determining the dimensions of tendon and also for the imaging of tendon morphology. Techniques used in clinical practice include magnetic resonance imaging, ultrasonography, and computed tomography. In addition, three-dimensional imaging is useful in the development of computational models for further prediction of material parameters using the inverse approach [30]. There have been many studies relating to 3D imaging techniques. However, there are no reliable standard methods for analyzing the geometry of wet, soft tissue samples directly prior to mechanical testing, which would ensure no changes in the boundary conditions, as well as the detection of the surface concavities.

Accurate measurements of biological sample geometry are of critical importance, especially for those intended for mechanical tests and further inverse analyses. The assumption that a specimen has a constant cross-section throughout its length can lead to fatal errors when estimating the parameters of material models or even make calibration impossible. Moreover, performing an inverse analysis of soft tissue strength tests requires not only the geometry of the sample, but also an accurate mapping of sample fixation in which the finite element method is performed using boundary conditions. Ensuring an unchanged sample fixation and, thus, boundary conditions between geometry imaging and mechanical tests (usually performed on different tests stands) is impossible. Because of this, there is a need to develop a rapid and reliable scanning system that is able to provide the exact geometry of the sample mounted in the tensile grips. The development of such a system would allow an inverse analysis of the mechanical test and extraction of parameters for both simple, as well as highly sophisticated material models [9,31,32,33,34,35].

The objective of our work was to develop a novel, fully automated, and reliable non-contact scanning system for 3D geometry determination of moist biological samples prior to carrying out mechanical tests subsequently used for inverse analyses. We used a triangulation-based blue light laser profile sensor, which allowed us to create computational models of the highly absorbing and reflective irregular tissue samples mounted in the tensile grips with ±2 μm accuracy and a relative error of less than 1.2%. Moreover, samples were mounted on replaceable inserts, which were fitted to the 3D scanning system, as well as the tensile machine. This ensured no changes in the boundary conditions between 3D scanning and mechanical tests, making inverse analysis feasible.

## 2. Development of a 3D Scanning System for the Imaging of Tendon Fascicle Bundles

The tissues of interest were soft and sensitive to moisture level. Because of this, the development of our scanning system had to meet several conditions. Three-dimensional analysis of the moist, soft tissue sample mounted in the grips, which would subsequently be subjected to tensile loading, required the completion of a 360–degree scanning angle in less than a few minutes to minimize sample dehydration prior to mechanical tests. Moreover, due to the moisture content and sample texture, our system should be resistant to light reflections and have high sensitivity, which would allow the detection of surface concavities. In this work, we propose a novel architecture of the 3D scanning system with the use of a blue light laser profile sensor and dedicated mechatronic system, which allowed us to obtain the 3D geometry of moist soft tissue samples. The proposed solution allowed for quick reconstruction of the sample geometry and ensured that boundary conditions remained unchanged during sample scanning, as well as during strength tests, which was of a critical importance for further inverse analysis.

### 2.1. Three-Dimensional Scanning System: Design

**Figure 1:**
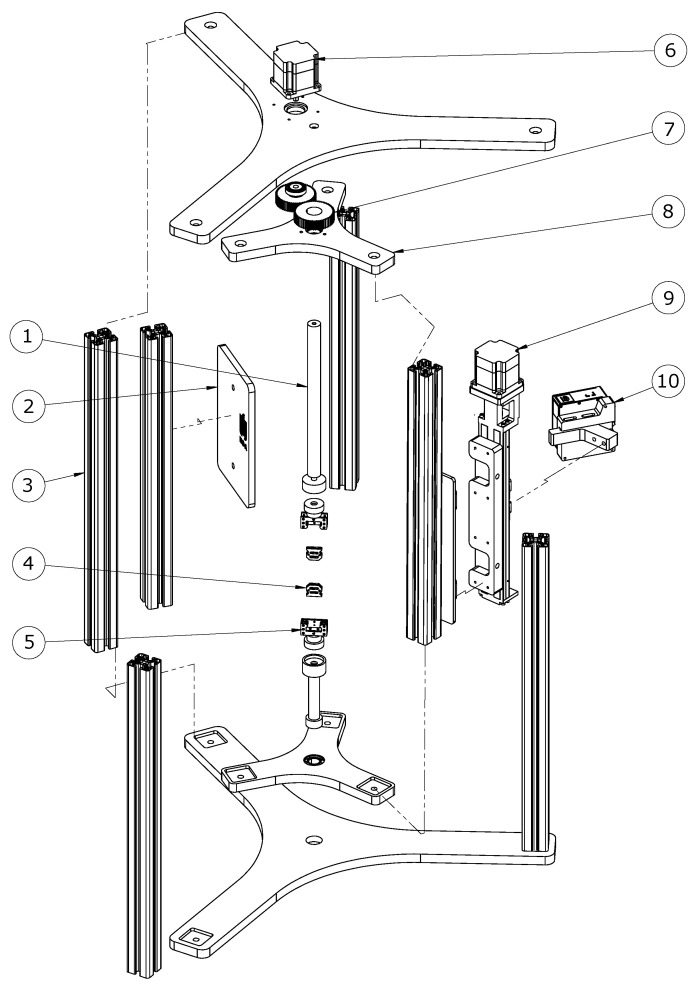
Assembly of the 3D scanner system: exploded view. The mechanical component is composed of: 1. adjustable clamping rod with neodymium magnet, 2. laser light protection plate, 3. aluminum profiles, 4. replaceable inserts for fixing samples, 5. tensile grips mounted with the sample onto the strength machine, 6. step motor for rotating the scanner, 7. drive gears, 8. revolving frame, 9. stepper motor controlling the vertical movement of the scanner, 10. blue laser profile sensor.

The 3D scanning system proposed in this study was composed of a Micro-Epsilon scanControl 2900–25/BL blue light laser profile sensor (Ortenburg, Germany), rotating custom-built frame (Figure 1), and associated software.

**Figure 2:**
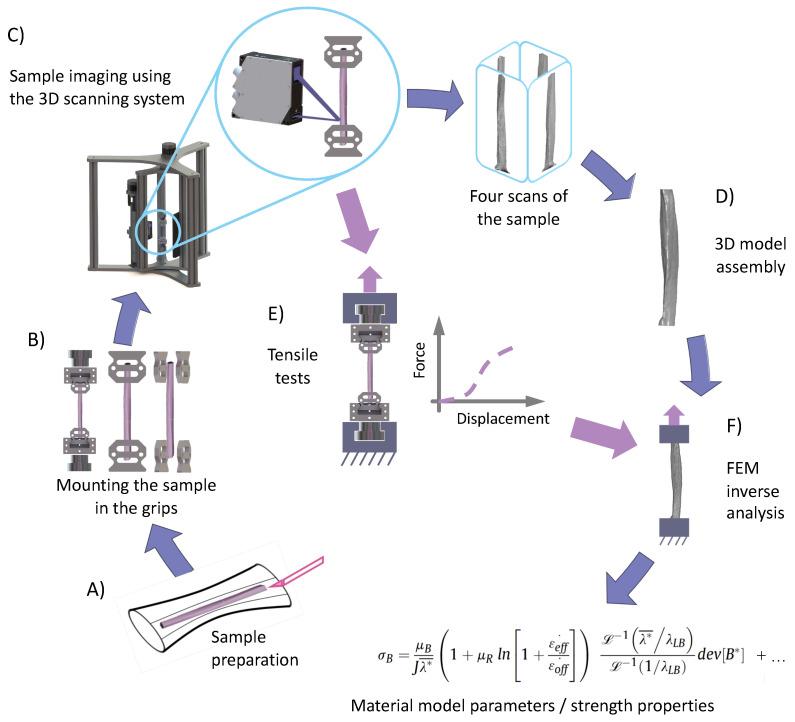
Flow diagram depicting the experimental process starting from the preparation of the samples (**A**), through mounting of the sample in replaceable inserts (**B**), scanning of the sample using the 3D scanning system (**C**), development of the 3D CAD model (**D**), up to the estimation of material model parameters using finite element method inverse analysis (**F**) based on force-displacement curves from tensile tests (**E**). Tensile tests were performed using the same gripping inserts that were used during 3D scanning of the sample, without changing the boundary conditions.

The scanControl 2900–25/BL blue light laser profile sensor had a resolution of 2 μm and a scanner sampling frequency of 300 Hz. The non-contact sensor used the triangulation method, which is commonly used to examine the shape of objects [36,37,38,39]. The blue light sensor used a laser diode with a wavelength of 405 nm and was suitable for transparent, semitransparent, and organic materials. The profile sensor was mounted onto a HIWIN KK 5002P300 single-axis robot (Offenburg, HIWIN, Germany) attached to the custom-made rotatory frame, which ensured rotation of the single-axis robot with the sensor around the stationary sample. Rotation of the frame and single-axis robot was driven by Oriental Motor PK566AW (Tokyo, Oriental Motor, Japan) five-phase stepper motors with a 0.72° step. One motor attached to the single-axis robot ensured movement of the profile sensor along the specimen, while a second motor controlled the rotation of the sensor, giving a 360° view of the sample. The motors were controlled by an Oriental Motor RKD514L-C (Tokyo, Oriental Motor, Japan) five-phase stepping motor unit. This motor unit offered a smooth drive function, which ensured low noise and low vibration even during low-speed operation. The smooth drive function automatically divided the step angle into 16 microsteps, so that changing the pulse signal was unnecessary. The motor unit was controlled by one-pulse input mode. To control the 3D scanning system, we used the Arduino microcontroller, an open-source electronics platform, which allowed the interaction with electronic devices, as well as the creation of a graphical user interface (GUI). The GUI was created using Processing software (www.processing.org), which is a Java-based open-source graphical library and integrated development environment. The GUI created for our 3D scanning system helped us to choose the number of scans rapidly and to control and calibrate its position. To obtain the 3D model of the sample, four scans were performed per 90-degree arc. The number of scans could be changed if required; however, four scans were sufficient to obtain accurate sample geometry and CSA cross-sectional measurement. Our proposed 3D scanner system enabled the measurement of samples with a diameter of less than 15 mm, with an accuracy of ±2 μm. The sensor position was controlled by an Infineon Technologies AG TLE4905L unipolar Hall switch (München, Infineon Technologies, Germany). The sample was mounted in tensile grips, attached to the scanning system by a neodymium magnet (Figure 2). The choice of all components was dictated by the need to create a novel and reliable 3D scanning system, which was easy to reconstruct by using existing elements. All components of our mechatronic system, with the exception of mechanical parts, were commercially available, which made the proposed solution easy to reproduce for other research groups. The blue light laser profilometer was designed for highly reflective and highly absorbent materials, which was crucial for moist biological materials.

Tensile grips were designed to be easily installed onto the 3D scanning system and to be quickly transferred to the strength machine without changing the boundary condition of the sample. Four 3D profiles were subsequently used for developing a fully 3D model of the sample using MeshLab software (www.meshlab.net) [40]. This process involved importing four sample scans and the rotation of the 3D profiles around the Y-axis every 90°. Next, we developed the Poisson surface from the cloud of points and generated the tetragonal mesh for further computational analysis.

### 2.2. Validation Procedure of Our 3D Scanning System on Synthetic Regular Samples and a Wooden Twig

In order to validate and determine the accuracy and reliability of our 3D scanner system, we scanned six samples (n = 6) composed of styrene acrylonitrile resin (SAN) having a cylindrical or hexagonal prism shape and then compared the results with two other methods: manual measurement with a caliper and a vision-based method. To evaluate the accuracy and reliability of our system for irregularly-shaped biological samples, we performed six measurements of a wooden twig. The dimensions of each sample were measured using a precision digital caliper with an accuracy of ±0.01 mm. The vision-based method consisted of taking photos of the analyzed sample in the presence of a caliper. Next, using Levenhuk ToupView software (Levenhuk USA), calibration was performed by determining dimensions from the scale of the caliper. Then, the sample dimensions (between the sample edges) were determined in several places. The following types of samples were included: a cylinder with a diameter of 9.46 ± 0.04 mm (Sample 1), a cylinder with a diameter of 8.98 ± 0.04 mm (Sample 2), a cylinder with a diameter of 2.32 ± 0.05 mm (Sample 3), a hexagonal prism with a short diagonal of 8.06 ± 0.05 mm (Sample 4), a hexagonal prism with a short diagonal of 6.61 ± 0.01 mm (Sample 5), a hexagonal prism with a short diagonal of 3.34 ± 0.03 mm (Sample 6), and a wooden twig with an irregular shape. Subsequently, six measurements were carried out for each cross-section, with the mean value and standard deviation recorded to assess for accuracy and repeatability of the 3D imaging technique. Sample scanning from four sides took approximately three minutes. The values of CSA, calculated based on the obtained 3D models, were compared to the estimated values based on caliper-, as well as vision-based measurements. Moreover, we estimated the relative error of the measurements to be the difference between the CSA value obtained by the evaluated method and the true value, divided by the true value. The true value refers to calculations based on measurements using a digital caliper.

### 2.3. Validation Procedure of Our 3D Scanning System on Soft Tissues: Tendon Fascicle Bundles

We used our 3D scanning system to image 10 samples dissected from Y-shaped bovine superficial digital flexor tendons (SDFT) obtained from a local abattoir. Immediately after receiving the fresh tendons, they were properly secured using moistened gauze, then frozen and stored at 80 °C. On the day of testing, each sample was slowly thawed for four hours at 4 °C and then for two hours at room temperature (23 ± 1 °C). After thawing, we removed two periphery branches of each tendon, leaving the core branch intact. Then, from the midsubstance of each core, we dissected a sample of fascicle bundles, ensuring that it did not include any piece of the tendon outer membrane. During cutting, we also guided the blade in such a way that the cut followed the natural torsion line of the fibers. The resulting cross-sectional area (CSA) of the fascicle bundle samples varied due to the unequal dimensions of the tendons from which they were cut. Despite the fact that the CSAs of our samples were approximately 15–30 mm^2^, we considered our samples to be fascicle bundles and not subtendons. This was because they were not dissected as an anatomically distinct portion of the multi-muscle tendon. Our 3D scanning system was used to create 3D models, as well as to calculate the CSA of each sample. The CSA values were calculated 3 mm above the lower grip. To prevent tissue dehydration, the samples were regularly sprayed between scans using a 0.9% NaCl solution. To investigate the influence of light reflections on scan reliability, we performed a repeat scan of all prepared tendon/tissue samples after coating them with talc. The application of a thin talc layer is a widely recognized method of reducing excessive laser light absorption or reflection. All tendon fascicle bundle samples were rinsed with a 0.9% NaCl solution and coated with talc before measurements. The measured CSA using our 3D scanning system was compared with the results obtained by the vision-based system. All statistical analyses were performed using OriginLab (OriginLab Corporation, Northampton, MA, USA). Values are presented as the mean ± standard deviation. The effect of the measurement method on the determined CSA was examined using a one-way analysis of variance (ANOVA) test. Tukey’s post-hoc analysis was used to determine significant differences between the mean values. The significance level was set to 0.05.

## 3. Results

### 3.1. Regular Cylindrical/Hexagonal Prismatic Shapes and Irregular Biological Samples: Comparison between the True Value, Vision-Based Method, and 3D Scanning System

The calculated CSA of the cylindrical and hexagonal prism SAN samples with standard deviations and relative errors for caliper measurements, our scanning system, and vision-based methods are presented in Table 1.

The standard deviation for CSA measured using our 3D scanning system for each sample was 0.02–0.06 mm^2^. The relative error for all samples measured using our 3D scanning system was less than 0.5%, while the vision-based method yielded a relative error between 4 and 8%. For prismatic specimens of regular hexagonal cross-sections, the angles between two sides when measured based on the reconstructed 3D model were: 120.1 ± 0.1° for Sample 4, 120.0 ± 0.0° for Sample 5, and 120.1 ± 0.1° for Sample 6, which were in line with their real values. Our 3D scanner system was also characterized by good repeatability. Coefficients of variation were less than 1.5% for all types of objects. The ANOVA test revealed statistically significant differences between the three measurement methods for all analyzed samples (*p* < 0.05). Tukey’s post-hoc analysis revealed that the values obtained by the vision-based method significantly differed from those obtained by 3D scanner and caliper measurement. After validation with regular specimens, we tested our 3D scanning system on a sample of a wooden twig. The calculated CSA of the wooden twig at several points with standard deviations and relative errors for caliper measurements, our scanning system, and vision-based methods are presented in Table 2.

The standard deviation for CSA measured using our 3D scanning system for each sample was 0.16–0.51 mm^2^. The relative error for all samples measured using our 3D scanning system was less than 1.2%, while for the vision-based method, the value of relative error was between 11 and 33%. The significant measurement error obtained by means of the vision-based method, resulting in overestimation of the CSA, may have been caused by image distortion from the lenses or chromatic aberration due to the imaging of samples against a contrasting background.

### 3.2. Tissue Samples: Comparison between the Vision-Based Method and 3D Scanning System Using Talc-Coated and Uncoated Samples

**Figure 3:**
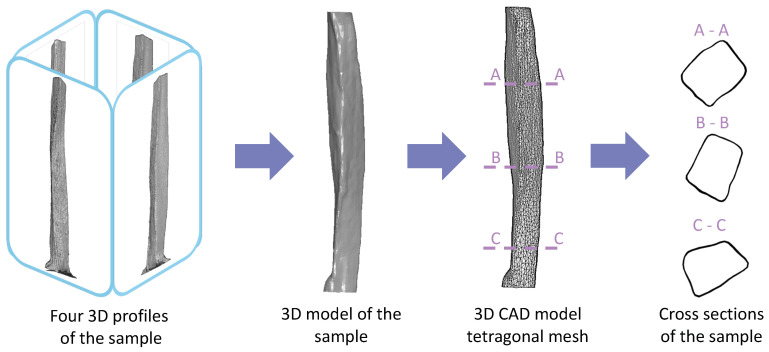
Development of the 3D model of the sample from four 3D profiles scanned every 90°. The profiles were assembled into a single 3D surface model considering concavities, followed by the generation of the tetragonal mesh for further computational strength analysis. Geometry of the sample with a variable cross-section is shown.

To evaluate the reliability of the 3D scanning system that we developed, we compared the CSA estimated using the vision-based method with CSA estimated based on 3D models, which is schematically shown in Figure 3. Moreover, we compared the results of vision-based measurements and those obtained via reconstructed 3D models with repeated measurements of fascicle bundles with additional anti-reflective talc coating [41]. The calculated CSA and relative error for CSA measurement of uncoated samples are presented in Table 3.

For tendon fascicle bundle samples, the 3D model of uncoated and talc-coated samples created using our 3D scanning system were compared with vision-based measurements. The use of calipers for the tactile measurement of tendon fascicle bundle dimensions was burdened by the large influence of operator experience and ability, which resulted in a measurement method of low repeatability. In this case, caliper measurements could not be considered as a reference measurement. The vision-based method overestimated the values of CSA, which was in line with our observations for regularly-shaped specimens. This overestimation could reach up to 31%, which resulted from sample concavity not being taken into consideration when using the vision-based method. Moreover, the significant error from vision-based measurements may have been caused by image distortion from the lenses or chromatic aberration due to the imaging of samples against a contrasting background. The application of talc coating to the sample, even though it could reduce laser light reflection, was not suitable in this situation due to the spread of the relative error value, reaching up to 22% (Table 3). This may be due to the uneven thickness of the talcum layer. The application of talc coating to tendon fascicle bundles did not increase the quality of the obtained 3D models; thus, it may be omitted. Our observations regarding the sample coating were in line with the results from a previous study [17]. The repeatability of measurements was high, giving coefficients of variation of less than 4% for both coated and uncoated tendons. The higher coefficients of variation obtained for tissue samples in comparison with regularly-shaped samples could result from the slight drying of soft tissues during measurements.

The accurate measurement of the sample geometry, especially of those intended for mechanical tests, is crucial for experimental research in the field of tissue biomechanics. A deep insight into the geometry of samples intended for mechanical tests is a key issue for experimental research in the field of tissue biomechanics. The influence of specimen dimensions on tendon mechanical properties has previously been studied [42,43]. However, these studies only considered the correlation between tissue mechanical properties and sample length. The correlation between mechanical properties and specimen CSA was described in detail by Legerotz et al. [44], who confirmed that failure stress, failure strain, and Young’s modulus positively correlated with CSA for a selected length of specimens. Therefore, the proper determination of CSA may be crucial for further calculations of selected mechanical properties of a sample, since overestimation of CSA results in underestimated strength properties of the samples. During previous studies, we observed that the mechanical response of tendons depended on sample geometry more than material properties, which was also reported by other research groups [45]. Therefore, the exact geometry of the sample allowed us to calculate the strength properties of biological samples and to estimate the parameters of the material models, which are crucial in cases of more sophisticated models. Commonly used techniques for measuring the geometry of tissue samples include the use of calipers [46,47], laser micrometers [48], or vision-based methods (camera snapshots, photos) [29]. Although the caliper method seems to be the simplest solution, it is largely based on operator decisions and the arbitrary selection of the first moment of contact between the instrument and tissue sample. This is a disadvantage of the technique, especially in the case of operators having little experience in the field of soft tissue experimentation. Furthermore, this may cause unintentional underestimation of CSA as a result of excessive tissue squeeze. This in turn may be insidious in the case of operators having little experience in the soft tissue experimental field and may cause unintentional underestimation of CSA as a result of excessive tissue squeeze. However, this issue can be overcome by performing a high number of repeated measurements by several different operators. Another approach for CSA measurement uses a laser micrometer, which is less dependent on intra- and inter-operator variability. However, the area detected by the laser beam does not take into consideration any concavities found on the tissue surface; thus, the detected CSA may not be the smallest along the sample’s length. This could lead to false estimations of tissue mechanical properties and incorrect research conclusions. An overestimation of CSA may also appear when using vision-based methods due to image distortion or chromatic aberration. Taken together, all of the above arguments suggest that the use of a manual or semi-automated solution for CSA measurement may never be as accurate as that of a fully automated system, which we have proposed in this study. However, in the case of experiments studying the influence of a given phenomenon on selected mechanical properties, an error resulting from measurements using vision-based or manual methods may be assumed as systematic error. In the absence of other factors that may cause gross or random errors, a systematic inaccuracy in CSA measurement may be identified as a constant offset factor. This should not have an influence on the relative change in mechanical parameters caused by the tested phenomena between groups. Moreover, the use of various methods for CSA measurement of soft tissue samples and the resulting variations in their values may explain the discrepancies in results from mechanical tests of tissues reported by different research groups.

The high repeatability and accuracy of our 3D scanning system on regularly-shaped, as well as tendon fascicle bundle samples demonstrated that this system could be successfully applied in the development of tissue 3D models, which could then be used for inverse analysis, as proposed in Figure 2. The scan acquisition time was approximately three minutes, which meant that this method was appropriate for fast drying biological samples. Furthermore, the 3D scanning system that we developed allowed non-contact detection of sample concavities due to the use of a laser profile sensor. The accuracy of the blue light profile sensor used in this study was equal to ±2 μm, while much more expensive structured light scanners, which are capable of capturing concavities [17], have an accuracy of 0.05 mm, which can be insufficient for the imaging of individual fascicle bundles or fascicles. The 3D scanners based on CCD laser displacement sensors [19,20] take into account sample concavities as well, but have an accuracy that is two-fold lower than our blue light sensor. Due to its short wavelength (405 nm), blue laser light did not penetrate the target surface, providing stable and precise results. Moreover, our developed 3D scanning system ensured that the boundary conditions remained unchanged during sample scanning, as well as during strength tests, which was crucial for the reconstruction of the models for further inverse analyses. Reconstruction of the sample surface from a cloud of points was performed using Poisson reconstruction in MeshLab. This software uses a portion of code written by Kazdan et al. [38] to build a hole-free surface, while filling in all the gaps. Our 3D scanning system had some limitations. The use of a laser triangulation sensor resulted in the appearance of a shadowing effect due to the distance between the receiver and laser beam transmitter. The shapes of grips and inserts, despite being optimized to limit the shadowing effect, did not allow accurate modeling of both sides of the sample together with both grips. A possible solution to overcome this issue might be to turn the laser sensor upside-down and to repeat the scanning process from the bottom, then assemble the scanned profiles together. Another alternative would be to use a second laser profile sensor simultaneously. The 3D scanner we proposed had numerous advantages, among others: non-contact fast measurement, high accuracy and repeatability, the ability to determine the dimensions of irregularly-shaped samples, as well as the quick transfer of the grips after scanning to the testing machine.

## 4. Conclusions

In our study, we described the development and validation of a novel 3D scanning system based on the blue laser profile sensor for the measurement of shapes and CSA of soft biological samples. Our proposed automatic 3D scanning system had the following noteworthy features:

- it used a blue laser profile sensor, which enabled the measurement of samples with a diameter of up to 15 mm with ±2 μm accuracy and a relative error of less than 1.2%
- it enabled the creation of 3D computational models of biological samples, including their concavities, within a matter of minutes
- samples were mounted on replaceable inserts, which were fitted to the 3D scanning system, as well as the tensile machine; this ensured no change in the boundary conditions between 3D scanning and mechanical tests, making inverse analysis feasible
- the process of sample scanning was fully automated, which ensured the high repeatability and accuracy of measurements

The validation of our 3D scanning system on regularly-shaped and tendon fascicle bundle samples showed that our non-contact scanning system could be successfully used in the development of 3D models, which is crucial for inverse analysis. Our system could be further developed by adding a second sensor turned upwards to eliminating the shadowing effect. Moreover, the 3D scanning system could be mounted directly onto the strength machine. Such a solution would allow for scanning of the sample shape under loading, which would allow for the estimation of the parameters in irregular specimen geometries. 

## Figures and Tables

**Table 1:** The results of cross-sectional area (CSA) measurements of the regular styrene acrylonitrile resin (SAN) samples and corresponding relative errors using our 3D scanning system and vision-based method. Number of repeated measurements per sample n = 6. Data concerning CSA are presented as means with standard deviations. Differences were considered statistically significant for *p* < 0.05.

Sample	True Value	3D Model		Vision-Based		
Number	CSA	CSA	Error	CSA	Error	
(Section)	(mm^2^)	(mm^2^)	(%)	(mm^2^)	(%)	
1 (circular)	9.46 ± 0.04	9.45 ± 0.02	0.06	10.02 ± 0.01	5.92	*p* < 0.001
2 (circular)	8.98 ± 0.04	9.02 ± 0.02	0.47	9.70 ± 0.09	8.00	*p* < 0.001
3 (circular)	2.32 ± 0.05	2.31 ± 0.02	0.13	2.47 ± 0.10	6.66	*p* < 0.001
4 (hexagonal)	8.06 ± 0.05	8.06 ± 0.02	0.07	8.44 ± 0.01	4.68	*p* < 0.001
5 (hexagonal)	6.61 ± 0.01	6.61 ± 0.06	0.01	6.90 ± 0.01	4.29	*p* < 0.001
6 (hexagonal)	3.34 ± 0.03	3.33 ± 0.02	0.30	3.37 ± 0.01	4.13	*p* < 0.05

**Table 2:** The results of CSA measurements of the biological sample (wooden twig) at 6 points of the sample and corresponding relative errors using our 3D scanning system and vision-based method. Number of repeated measurements per point n = 6. Data are presented as means with standard deviations.

Section	True Value	Reconstructed 3D Model		Vision-Based Method		
Number	CSA	CSA	Error	CSA	Error	
	(mm^2^)	(mm^2^)	(%)	(mm^2^)	(%)	
1	29.21	28.89 ± 0.16	1.12	35.56 ± 2.00	17.85	*p* < 0.001
2	34.51	34.82 ± 0.42	0.90	39.10 ± 1.66	11.74	*p* < 0.001
3	29.40	29.38 ± 0.51	0.06	44.01 ± 2.26	33.20	*p* < 0.001
4	33.99	33.89 ± 0.51	0.15	45.89 ± 0.67	25.86	*p* < 0.001
5	46.66	47.20 ± 0.18	1.13	67.99 ± 1.10	31.36	*p* < 0.001
6	30.27	29.94 ± 0.34	1.10	45.20 ± 0.82	33.03	*p* < 0.001

**Table 3:** The calculated cross-sectional areas (CSA) of tendon fascicle bundles estimated using the 3D scanning system for uncoated and talc-coated samples compared to vision-based measurements. Number of repeated measurements per sample n = 6. Data concerning CSA are presented as means with standard deviations.

Tendon	3D Model	3D Model		Vision-Based	
Fascicle	Uncoated	Talc-Coated	Error	CSA	Error
Bundle	CSA (mm^2^)	CSA (mm^2^)	(%)	(mm^2^)	(%)
1	22.97 ± 0.32	26.96 ± 0.69	17.37	24.85 ± 0.30	8.18
2	30.27 ± 0.36	34.82 ± 0.84	15.03	32.70 ± 0.32	8.00
3	26.05 ± 0.40	30.84 ± 0.93	18.39	28.42 ± 0.41	9.10
4	17.40 ± 0.28	20.95 ± 0.64	20.40	19.94 ± 0.28	14.60
5	20.57 ± 0.78	20.15 ± 0.73	−2.04	23.65 ± 0.25	14.97
6	24.00 ± 0.76	27.02 ± 0.30	12.58	29.84 ± 0.78	24.33
7	17.98 ± 0.21	21.78 ± 0.53	21.13	19.86 ± 0.34	10.46
8	24.57 ± 0.29	28.27 ± 0.51	15.06	32.10 ± 0.45	30.65
9	14.88 ± 0.54	16.26 ± 0.37	9.27	18.88 ± 0.52	26.88
10	16.23 ± 0.61	19.70 ± 0.41	21.38	18.10 ± 0.41	11.52
